# Time-Frequency Representation of Motor Evoked Potentials in Brain Tumor Patients

**DOI:** 10.3389/fneur.2020.633224

**Published:** 2021-02-05

**Authors:** Kathrin Machetanz, Alberto L. Gallotti, Maria Teresa Leao Tatagiba, Marina Liebsch, Leonidas Trakolis, Sophie Wang, Marcos Tatagiba, Alireza Gharabaghi, Georgios Naros

**Affiliations:** ^1^Neurosurgical Clinic, Department of Neurosurgery and Neurotechnology, Eberhard Karls University of Tuebingen, Tuebingen, Germany; ^2^Department of Neurosurgery and Neurotechnology, Institute for Neuromodulation and Neurotechnology, Eberhard Karls University of Tuebingen, Tuebingen, Germany; ^3^Department of Neurosurgery and Stereotactic Radiosurgery, Vita-Salute University, Milan, Italy

**Keywords:** transcranial magnetic stimulation, motor evoked potentials, brain tumors, time-frequency analysis, frequency domain, inter-trial coherence

## Abstract

**Background:** The integrity of the motor system can be examined by applying navigated transcranial magnetic stimulation (nTMS) to the cortex. The corresponding motor-evoked potentials (MEPs) in the target muscles are mirroring the status of the human motor system, far beyond corticospinal integrity. Commonly used time domain features of MEPs (e.g., peak-to-peak amplitudes and onset latencies) exert a high inter-subject and intra-subject variability. Frequency domain analysis might help to resolve or quantify disease-related MEP changes, e.g., in brain tumor patients. The aim of the present study was to describe the time-frequency representation of MEPs in brain tumor patients, its relation to clinical and imaging findings, and the differences to healthy subject.

**Methods:** This prospective study compared 12 healthy subjects with 12 consecutive brain tumor patients (with and without a paresis) applying nTMS mapping. Resulting MEPs were evaluated in the time series domain (i.e., amplitudes and latencies). After transformation into the frequency domain using a Morlet wavelet approach, event-related spectral perturbation (ERSP), and inter-trial coherence (ITC) were calculated and compared to diffusion tensor imaging (DTI) results.

**Results:** There were no significant differences in the time series characteristics between groups. MEPs were projecting to a frequency band between 30 and 300 Hz with a local maximum around 100 Hz for both healthy subjects and patients. However, there was ERSP reduction for higher frequencies (>100 Hz) in patients in contrast to healthy subjects. This deceleration was mirrored in an increase of the inter-peak MEP latencies. Patients with a paresis showed an additional disturbance in ITC in these frequencies. There was no correlation between the CST integrity (as measured by DTI) and the MEP parameters.

**Conclusion:** Time-frequency analysis may provide additional information above and beyond classical MEP time domain features and the status of the corticospinal system in brain tumor patients. This first evaluation indicates that brain tumors might affect cortical physiology and the responsiveness of the cortex to TMS resulting in a temporal dispersion of the corticospinal transmission.

## Introduction

The human motor system consists of several cortical, subcortical, and spinal hubs. For unobstructed voluntary movements corticospinal integrity is required. Cerebral lesions (e.g., stroke, brain tumors) can affect corticospinal transmission and impair voluntary movements (1). In the past years, there is a tremendous progress in evaluating the human motor system of these patients with electrophysiological means such as transcranial magnetic stimulation (TMS). The magnetic cortical input through TMS is suggested to activate excitatory and inhibitory neurons, transmitting their information in volleys (i.e., D- and I-waves) to the spinal cord and resulting in a synchronized activation of muscle cells, which can be measured as motor-evoked potentials (MEPs) (2–4). Cortical excitability and stimulation intensity determine the size of descending volleys and, hence, the amplitude of the MEP. The conduction time of neural impulses traveling along the cortico-spinal projections to peripheral muscles is reflected in the latency of the MEP (2–4). Thus, motor-evoked potentials are mirroring the status of the complete human motor system. In line, it has been shown that MEP characteristics are influenced by the current muscular (5, 6), spinal (7), and cortical status (6, 8).

Evoked potentials (EPs) such as MEPs are short phasic events, which are commonly evaluated in the time series domain of a single trial or after averaging over several trials. Temporal dispersion of the descending volleys changes latencies, shape, and amplitudes of the EP and impedes its interpretation (3). In fact, time domain features of MEPs are sensitive to noise and exert a high inter-subject and intra-subject variability (9–12). While an increase of the MEP latency and a decrease of the MEP amplitude are indicative of a lesion to the corticospinal network (13–15), little attention is paid to the exact shape of the MEP. However, electromagnetic signals can also be described in the frequency domain. While time-domain studies evaluate the signal fluctuation over time, frequency-domain analyses transform the signal into a sum of oscillations (i.e., sine waves) and describe the contribution of different frequencies to the complete signal (i.e., power). Despite losing some temporal information, the frequency domain perspective has several potential advantages enabling a description of EP shapes and allowing the application of further neuroscientific concepts (e.g., phase behavior or inter-trial coherence, ITC), which might help to resolve or quantify the temporal dispersion of EPs. While being ubiquitous in neuroscience (6, 16–18), frequency domain analysis techniques are infrequently found in the clinical setting. However, there is an increasing interest in the frequency representation of EPs in animals (19–24) and humans (25, 26).

The aim of the present study is to describe the time-frequency representation of MEPs in brain tumor patients, its relation to clinical and imaging findings, and the differences to healthy subjects. To the best of our knowledge, this is the first study evaluating MEPs of brain tumor patients in the frequency domain.

## Methods

### Patients

This prospective study covers 12 healthy subjects (30.2 ± 13.9 years, 10 female) and 12 consecutive patients (51.3 ± 20.3 years, nine female) with motor eloquent brain lesions who underwent an nTMS mapping in the Neurosurgical Department of the University of Tuebingen. Patients were classified into two categories by an experienced neurosurgeon based on their clinical motor status in the Medical Research Council Scale (MRCS): six patients had no motor signs (MRCS 0) and six patients showed an upper limb paresis (MRCS<5). Details of clinical and demographic characteristics of the patients are depicted in Table 1. The study was approved by the local ethics committee of the Eberhardt Karls University Tuebingen. All participants gave written informed consent.

**Table 1:** Patients' clinical, imaging, and electrophysiological characteristics.

	**Group 1** **healthy subjects**	**Group 2** **no motor signs**	**Group 3** **apparent paresis**	***p*-value**
	***n*** **= 12**	***n*** **= 6**	***n*** **= 6**	
Age	30.2 ± 13.9	39.2 ± 20.9	63.5 ± 10.6	**0.006**
Gender (f:m)	10:2	3:3	6:0	0.091
**Diagnosis**				
HGG	-	3	4	0.558
Metastasis	-	3	2	
**Tumor size (cm**3**)**	-	8.7 ± 6.3	12.5 ± 14.9	0.873
**DTI**				
Mean FA	-	0.43 ± 0.06	0.46 ± 0.03	0.749
Mean ADC (10^−4^ mm^2^/s)	-	8.86 ± 1.13	8.44 ± 3.44	0.522
**nTMS**				
RMT (%)	36 ± 6	37 ± 11	51 ± 8	**0.013**
No. of trials	226 ± 91	185 ± 85	199 ± 94	0.712
MEP + trials	90 ± 48	81 ± 57	155 ± 45	0.162
Amp (μV)	241 ± 185	160 ± 100	199 ± 132	0.827
Lat0 (ms)	23.6 ± 0.9	23.3 ± 2.5	22.8 ± 3.1	0.551
Lat1 (ms)	27.2 ± 0.8	27.3 ± 2.2	27.4 ± 3.5	0.614
Lat2 (ms)	31.4 ± 1.4	32.4 ± 1.7	32.8 ± 3.9	0.589
Lat3 (ms)	66.8 ± 16.2	59.9 ± 9.4	65.4 ± 12.2	0.906
Lat0-Lat1 (ms)	3.7 ± 0.5	4.0 ± 0.8	4.6 ± 1.0	0.103
Lat0-Lat2 (ms)	7.3 ± 0.7	9.1 ± 2.0	10.0 ± 2.8	**0.031**
Lat0-Lat3 (ms)	38.3 ± 13.3	36.6 ± 9.7	42.6 ± 12.5	0.608
Lat1-Lat2 (ms)	3.6 ± 0.5	5.1 ± 1.3	5.4 ± 2.7	**0.058**
Lat2-Lat3 (ms)	31.0 ± 13.3	27.6 ± 8.7	32.6 ± 12.8	0.898
ERSP1^*^	35.3 ± 6.6	31.3 ± 8.5	26.7 ± 10.1	0.158
ERSP2^**^	28.8 ± 7.4	28.5 ± 9.4	20.9 ± 9.8	0.221
ITC^*^	0.64 ± 0.15	0.74 ± 0.05	0.43 ± 0.07	**0.006**
ITC^**^	0.56 ± 0.16	0.73 ± 0.02	0.36 ± 0.10	**0.002**

* *for 20–30 ms and 40–200 Hz*.

** *for 30–40 ms and 40–200 Hz. Significant p-values are depicted in bold letters*.

### Magnetic Resonance Imaging (MRI)

All healthy subjects and patients received preoperative MR imaging using a 1.5 T MR imaging unit (Skyra/Prisma-fit/Aera, Siemens Healthineers, Erlangen, Germany) with an 8-channel head coil. Patients received T1-weighted (contrast-enhanced) echo sequences and, additionally, diffusion tensor imaging (DTI). DTI was performed with a single-shot spin echo at a *b*-value of 1,000 s/mm^2^ along 12–64 geometric directions. Following, the anatomical MRI data set was imported to our nTMS system (Nexstim Eximia, version 3.2.2, Helsinki, Finland) for further data acquisition and analysis.

### Navigated Transcranial Magnetic Stimulation (nTMS)

**Figure 1:**
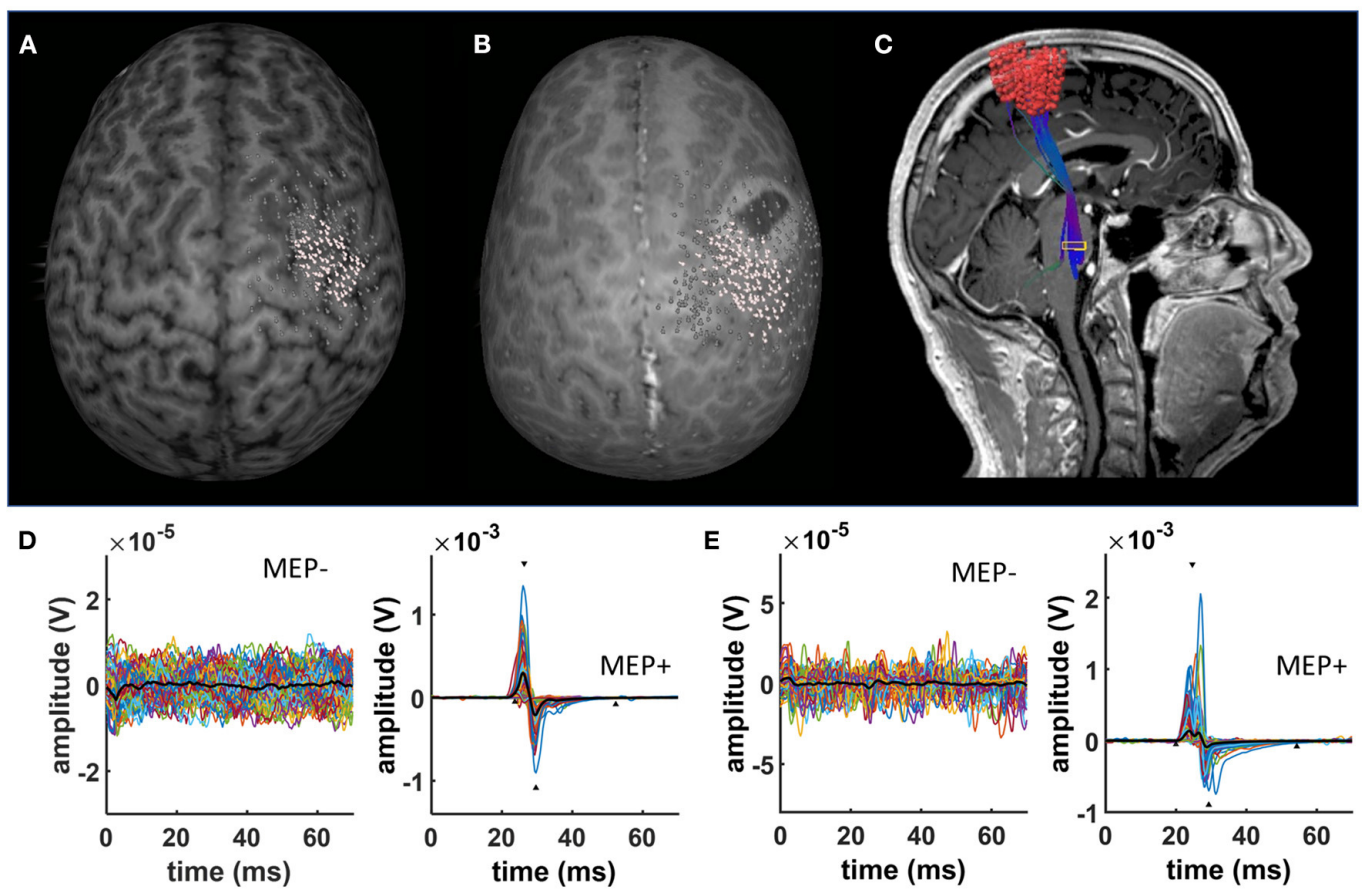
nTMS results. Exemplary data of a characteristic nTMS map in **(A)** a healthy subject and **(B)** a patient with a brain tumor. White dots represent nTMS coordinates eliciting a MEP. In contrast, gray dots indicate spots with no MEPs. nTMS results in patients were used as a seed for deterministic DTI fiber tracking **(C)**. **(D)** Shows exemplary EMG data of a healthy subject separating trials without (MEP-) and with MEPs (MEP+). **(E)** Shows exemplary EMG data of a brain tumor patient separating trials without (MEP-) and with MEPs (MEP+).

The cortical mapping procedure was described previously and is applied here in the same way (27–30): We used a navigated TMS stimulator (eXimia®, Nexstim, Helsinki, Finland) and a biphasic figure-8 coil. Prior to the mapping, patients' anatomical T1-weighted magnetic resonance images were co-registered to the patient's head with a registration error of <2 mm. After determining the “hotspot” yielding the largest motor-evoked potential (MEP) from the contralateral abductor pollicis brevis muscle (APB), the resting motor threshold (RMT), defined as the minimum stimulus intensity to result in at least 5/10 trials a MEP > 50 μV, was obtained. The orientation of the induced current in the brain was posterior-anterior for the first phase and anterior-posterior for the second phase of the stimulus. The orientation of the electric field, calculated on the basis of the individual MRI of each subject by the eXimia software, was kept perpendicular to the central sulcus. Subsequently, the cortex was mapped with 110% RMT starting at the primary motor cortex and then extending around this spot to cover the primary motor cortex, somatosensory cortex, and premotor cortex (Figures 1A,B). Thus, an average of 209.2 ± 8.3 [96–394] stimuli were applied per patient and map. Stimulation sites were visualized on the surface at a depth of 25–30 mm. Coordinates of the stimulation sites were automatically saved by the eXimia software for later analysis (e.g., DTI-based tractographie, Figure 1C).

### Electromyographic Recordings (EMG)

During nTMS mapping, myoelectric signals of the contralesional abductor pollicis brevis (APB) and the first dorsal interosseous muscles (FDI) were recorded with the integrated EMG device of the eXimia system (3 kHz sampling rate, band-pass filter of 10–500 Hz) using Ag/AgCl wet gel surface electrodes (AmbuNeuroline 720, Ambu GmbH, Germany).

### EMG Data Analysis

Data analysis was performed using custom-written scripts in MATLAB (Mathworks Ltd, USA, R2017a), applying functions of the open source toolboxes EEGlab (31) and Fieldtrip (32). EMG data was imported into Matlab and segmented into epochs from −100 to 100 ms relative to the TMS pulse. No further data processing was performed except of linear detrending of the epochs. Generally, the APB muscle was selected for further analysis. The nTMS trials were classified in MEP+ and MEP- trials depending on a MEP amplitude (≥20 μV) and latency (≥15 and ≤ 30 ms) threshold (Figures 1D,E). Trials with artifacts or EMG pre-stimulus activation were automatically removed from further analysis. In case of a bad signal-to-noise ratio or a number of artifacts higher than the average, the FDI muscle was chosen for further analysis. A Matlab-based custom-written script was used to automatically detect several time series characteristics of the MEP: Amp (i.e., peak-to-peak amplitude), Lat0 (i.e., MEP onset latency), Lat1 (i.e., latency of the maximum positive deflection of the MEP), Lat2 (latency of the minimum negative deflection of the MEP), and Lat3 (i.e., ending of the MEP). The time-frequency analysis of the MEP was performed on the basis of a Morlet wavelet approach with a fixed wavelet length of 40 ms (as implemented by the *newtimef* function of the EEGlab toolbox) (31). The wavelet length was chosen considering the average length of a MEP (i.e., Lat3-Lat0) and represents a balance between power and the phase precision of the analysis (see *Discussion*). This approach resulted in a spectral resolution of 1 Hz (30–500 Hz) and temporal resolution of 0.333 ms (−79.333 to 79.333 ms relative to the TMS pulse). Event-related spectral perturbation (ERSP) was calculated (in dB) and trial-wise normalized to the baseline spectrum (−79.3 to −10 ms relative to the TMS pulse) to reduce sensitivity to noisy trials (33). The ITC measures event-related phase coherence across trials. It is obtained from the phase information in the spectral decomposition while normalizing the magnitude information. Hence, the ITC is an amplitude-independent measure for phase-locking. The ITC values represent the circular variance of phases (34) and range from 0 to 1, with a value of 1 being indicative of perfect phase-locking.

### MR Imaging Analysis

After nTMS mapping, the coordinates of MEP+ trials were exported as DICOM from the Nexstim software and imported into the BrainLab iPlan 3.0 software. A cortical ROI was constructed from the summation of MEP+ and enlarged by 2 mm (35, 36). The ROI was fused to the anatomical T1-weigthed MRI and DTI dataset. In addition to the cortical ROIs, a subcortical ROI was placed in the caudal pons based on the color-coded FA map (35–41). The corticospinal tract (CST) was detected using a fiber length of 110 mm and a FA value corresponding to 75% of the individual FA threshold impeding any fiber detection (35, 36, 42). Mean FA and ADC values of the resulting CST was noted as an imaging surrogate of its integrity. Additionally, BrainLab software was used to delineate the tumor extent and to determine its volume (in cm3).

### Statistics

Statistical evaluation was performed using SPSS (IBM SPSS Statistics for Windows, Version 25.0, Armonk, NY: IBM Corp.) and custom-written Matlab scripts including the FieldTrip toolbox and Matlab statistics toolbox. Group effects on clinical (age, gender, diagnosis), imaging (FA and ADC values), as well as electrophysiological characteristics (RMT, no. of trials, MEP amplitudes and latencies, ERSP and ITC values) were evaluated by non-parametric Kruskal-Wallis, Wilcoxon, and *X*^*2*^-tests when applicable. Correlation analyses between electrophysiological and clinical parameters were based on Pearson's correlations coefficients. Group differences in the time-frequency representation of the MEPs (ERSP and ITC) were assessed by an unpaired *t*-test. Multiple comparison correction was based on a non-parametric permutation test (200 permutations) as implemented in the FieldTrip toolbox. The *t-*values that exceeded an *a priori* threshold of *p* < 0.05 were subsequently clustered in connected sets based on temporal (i.e., time windows) and spectral parameters. Cluster-level statistics were then calculated by taking the sum of the *t-*values within every cluster and the resultant maximum summed *t*-values were used to compute the statistical comparisons. The significance probability was calculated using a Monte-Carlo method (43). By randomizing the data, the reference distribution of the maximum of summed cluster *t-*values was acquired to evaluate the actual data significance statistic. Clusters from the original data were considered to be significant (alpha level 5%) if <5% of the reference distribution permutations returned a maximum cluster-level statistic larger than the cluster-level value detected in the original data. This cluster-based approach was used to compare the MEP response between the different groups for ERSP and ITC. Results are shown as mean ± standard deviation (SD).

## Results

### Patients' Characteristics

The present study includes 12 healthy subjects (*Group 1*) and 12 patients with brain tumors who underwent nTMS brain mapping prior to brain surgery. Patients were classified into two categories based on their clinical motor status (*Group 2*: six patients no motor signs, MRCS 5; *Group 3*: six patients with an apparent upper limb paresis MRCS<5). There were no significant differences in gender distribution (*p* = 0.091; *X*^*2*^-test). Patients with an apparent paresis were significantly older than the other two groups (*p* = 0.006; Kruskal-Wallis); however, there were no significant age differences between the healthy subject group and the patient group without motor signs (*p* = 0.471; Wilcoxon). There were no significant differences in the distribution of tumor diagnosis or size between the patient groups (*p* = 0.588; *X*^2^-test and *p* = 0.873; Kruskal-Wallis). nTMS results of the patients (i.e., coordinates of positive responses) were used for corticospinal fiber tracking on the individual DTI scan. There were no significant differences in the mean FA and ADC values of the detected corticospinal tract (*p* = 0.749 and *p* = 0.522; Kruskal-Wallis). All results are summarized in Table 1.

### nTMS Time Series Results

**Figure 2:**
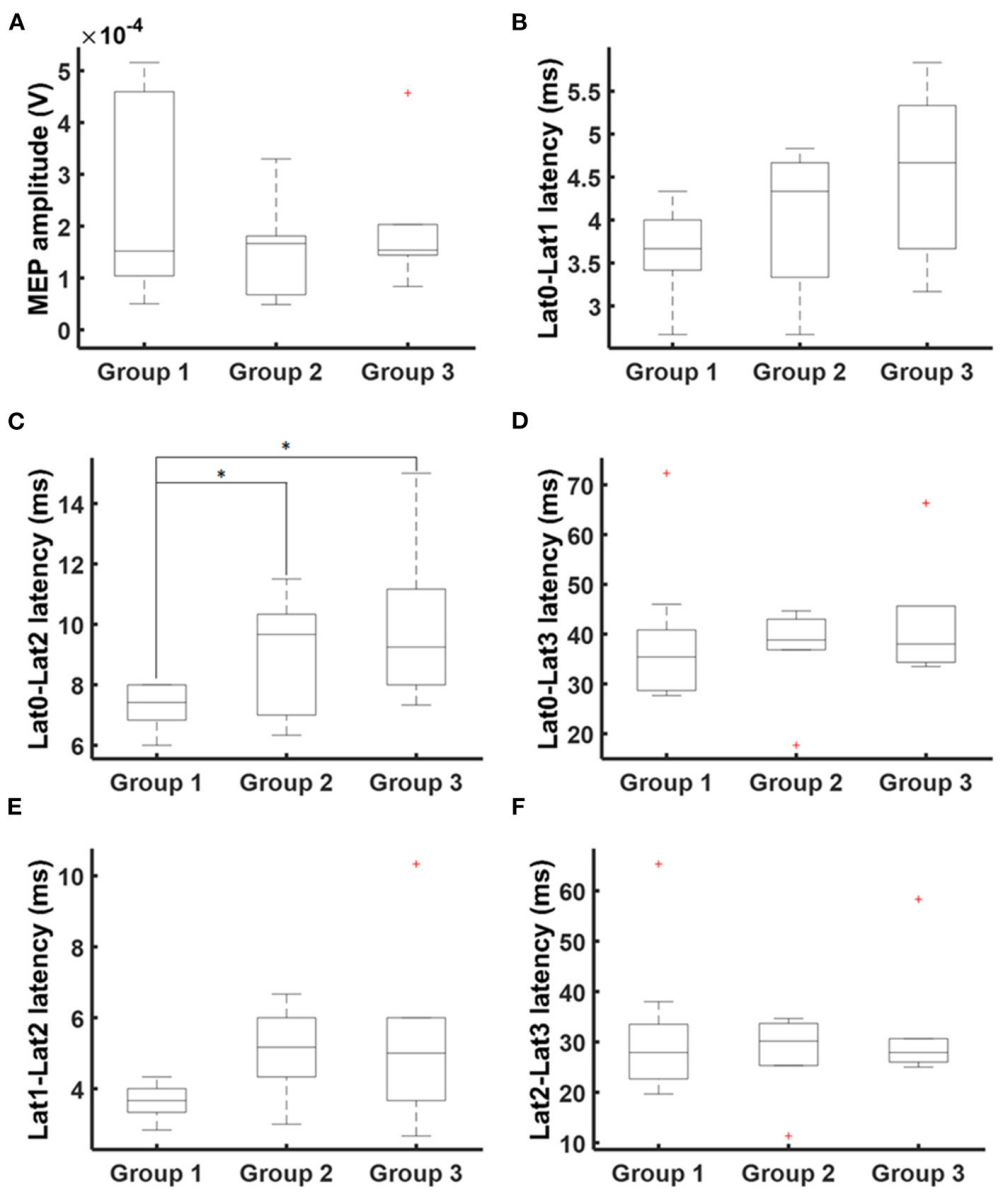
MEP times series characteristics. Times series analysis covered MEP amplitudes **(A)** and latencies Lat0 (MEP onset), Lat1 (maximum positive deflection), Lat2 (minimum negative deflection), and Lat3 (MEP ending). Additionally, latency differences were calculated: **(B)** Lat0-Lat1, **(C)** Lat0-Lat2, **(D)** Lat0-Lat3, **(E)** Lat1-Lat2, and **(F)** Lat2-Lat3. There was a deceleration of MEP. Statistical significance is marked with an asterisk (*p* < 0.05; Wilcoxon).

**Figure 3:**
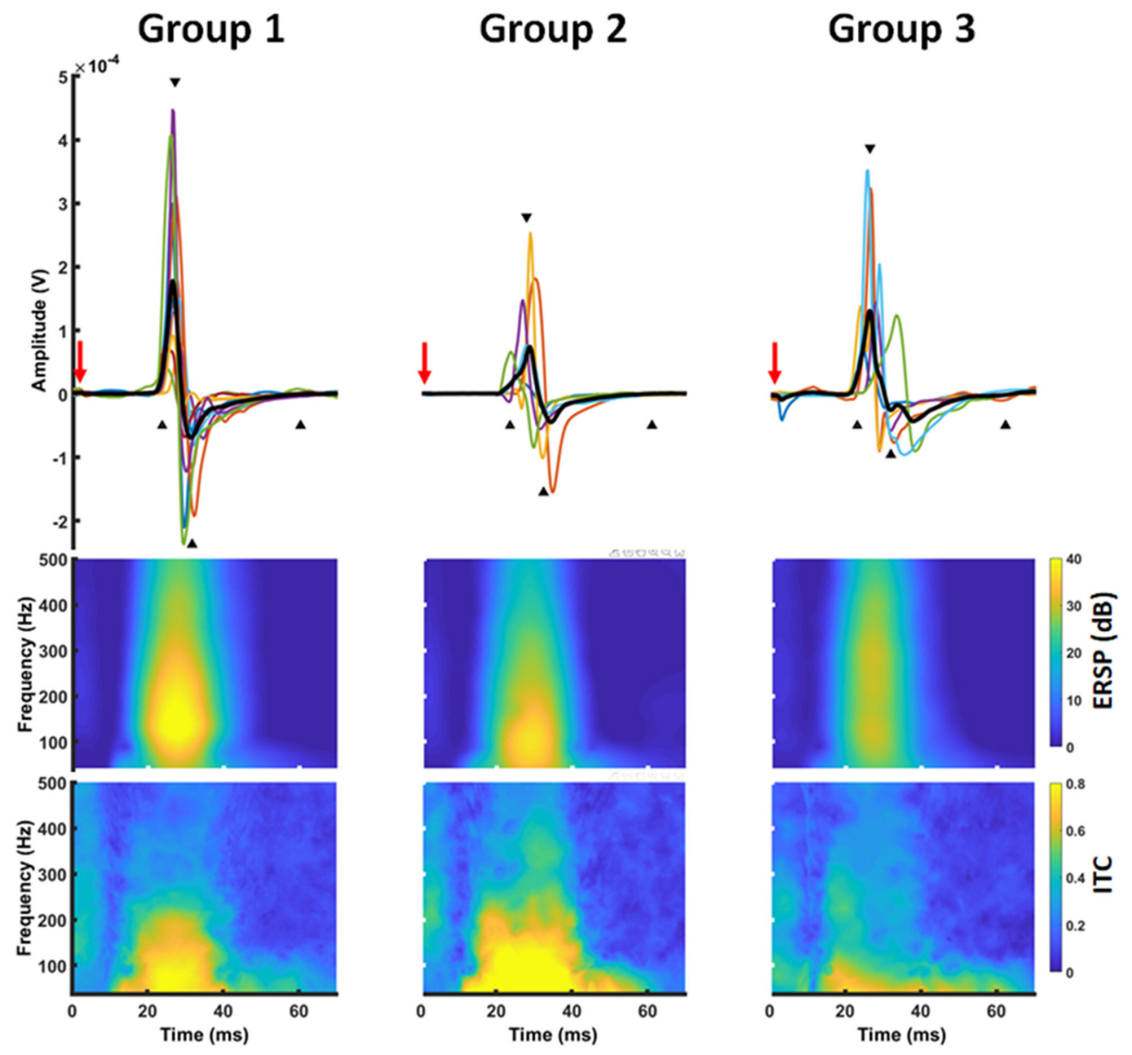
Time-frequency representation of a MEP. MEP time series **(upper row)** were transferred into the frequency domain using a Morlet wavelet approach. The transformation revealed a power increase (ERSP) ~20–50 ms after TMS application (red arrow) in a frequency band between 30 and 300 Hz with a local maximum around 100 Hz **(middle row)**. At the same time, there was a high inter-trial-coherence covering the frequency and time range **(lower row)**.

nTMS cortical mapping was performed in all healthy subjects and patients in a similar manner (exemplary data see Figure 1A). Patients with an apparent paresis (*Group 3*) had a significant higher resting motor threshold than healthy subjects (*Group 1*) and patients without motor signs (*Group 2, p* = 0.013; Kruskal-Wallis). There were no significant group differences in the number of applied TMS pulses (*p* = 0.712; Kruskal-Wallis). Notably, there was a higher variance of the MEP shape for patients than for healthy subjects (exemplary data see Figure 1B). There were no significant group differences of the mean MEP amplitudes (Table 1, Figure 2A) and the different latency measures (Table 1). Notably, we observed a deceleration of the MEP in the patient groups as documented by the differences between Lat0, Lat1, and Lat2 (Table 1, Figures 2, 3). There was no correlation between the time series characteristics (*p* > 0.05; Pearson's) and the age, tumor volume, ADC values, FA values, and RMT except of a significant positive correlation between the RMT and the latency Lat1-Lat2 (*r* = 423; *p* = 0.049).

### nTMS Time-Frequency Results

**Figure 4:**
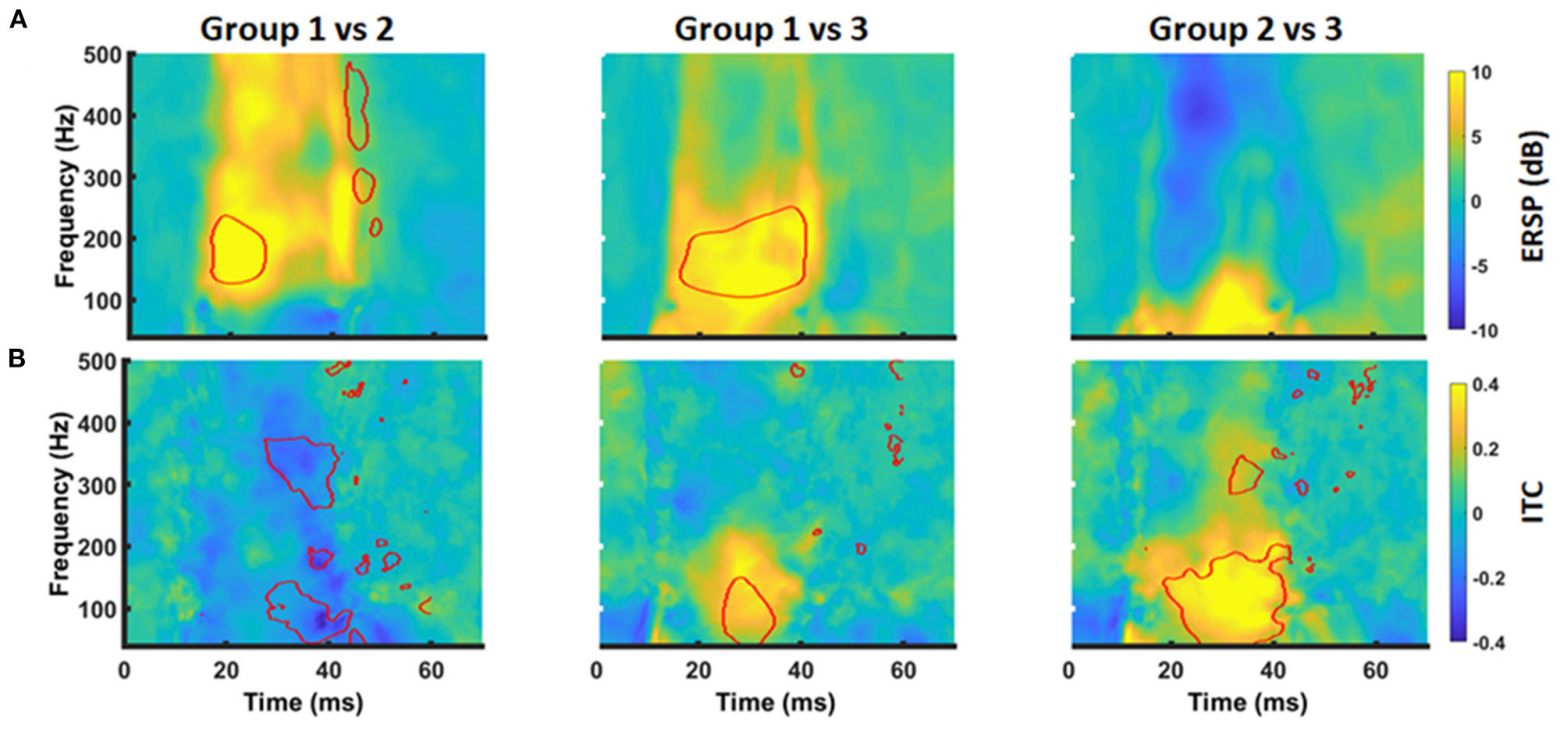
Group differences of time-frequency MEP behavior. Group differences in ERSP **(A)** and ITC **(B)** between Group 1 and 2 (*left column*), Group1 and 3 (*middle column*), and Group 2 and 3 (*right column*) were evaluated by cluster-based permutation analysis. Significant time-frequency bins are outlined in red (*p* < 0.05, cluster corrected).

MEP data was transferred into the frequency domain using a Morlet wavelet approach. The transformation revealed a projection of the MEPs to a frequency band between 30 and 300 Hz with a local maximum around 100 Hz. At the same time, there was a high ITC covering the same frequencies. Notably, this pattern was similar for all groups (Figure 3). Cluster-based significance analysis showed a significant power reduction between 100 and 200 Hz in a time period of 20–30 ms for patients without any motor signs in comparison to healthy subjects. Notably, there was no reduction of ITC in that period. In contrast, these patients showed an increased ITC during the later course of the MEP (i.e., 30–40 ms after TMS). Patients with a paresis, however, showed both a power reduction and a reduced ITC in comparison to healthy subjects during the whole MEP duration (i.e., 20–40 ms). When comparing brain tumor patients with and without paresis, we noticed no further power reduction. But there was a further disturbance of the ITC (Figure 4).

**Figure 5:**
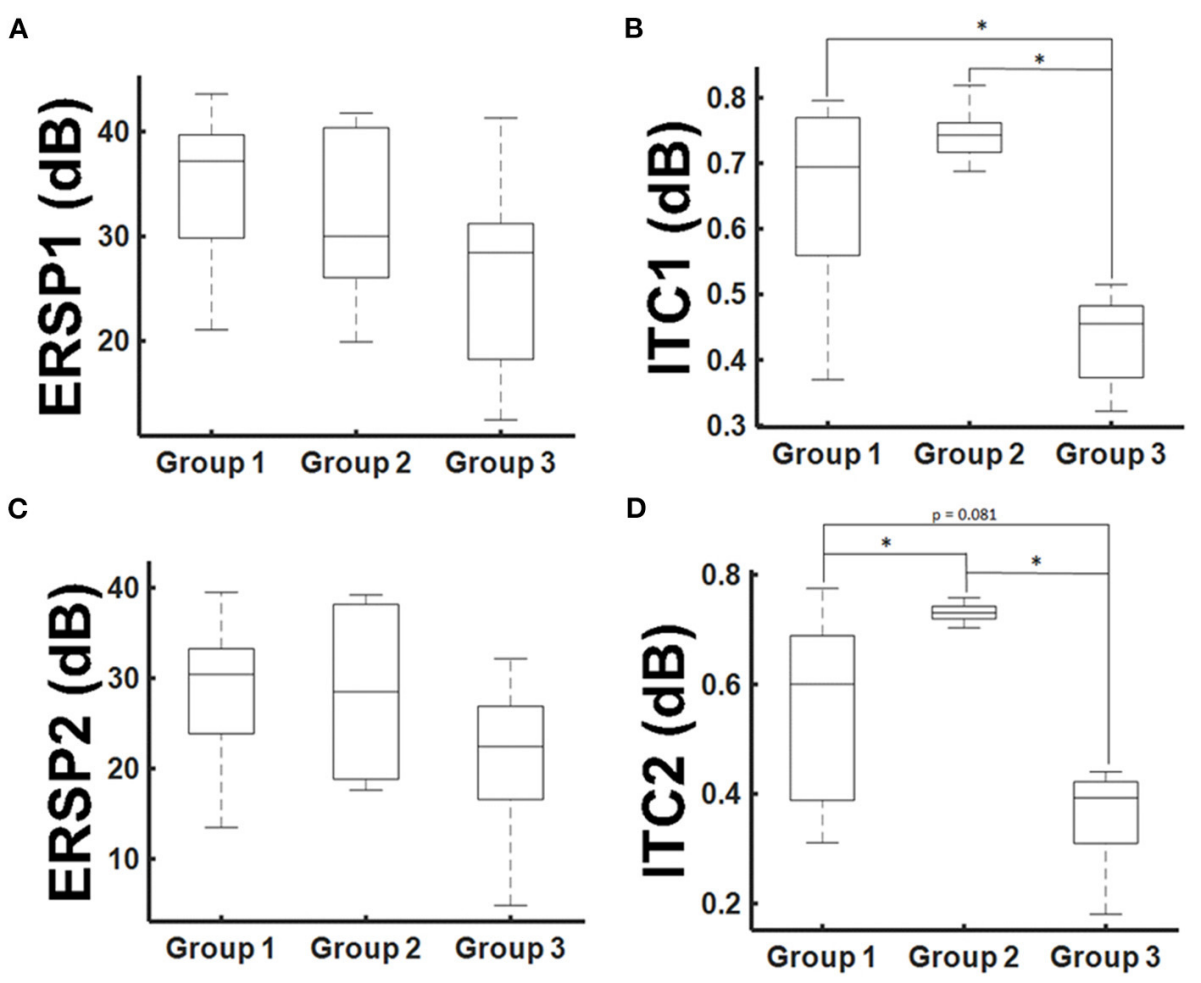
ERSP and ITC group results. Mean ERSP and ITC values for the 30–200 Hz frequencies averaged for the time period of 20–30 ms after TMS application [ERSP1 and ITC1, **(A,B)**] and 30–40 ms [ERSP2 and ITC2, **(C,D)**]. Statistical significance is marked with an asterisk (*p* < 0.05; Wilcoxon).

On the basis of these findings, we performed a secondary analysis of the mean ERSP and ITC averaging for the frequency band of 30–200 Hz and considering the time periods of 20–30 ms (ERSP1 and ITC1) and 30–40 ms (ERSP2 and ITC2), respectively. With this approach, ERSP findings were not significant anymore (Figures 5A,B; *p* > 0.05, Kruskal-Wallis). However, there was a significant group effect for the ITC1 and ITC2 (*p* = 0.006 and *p* = 0.002; Kruskal-Wallis). There was a significant ITC1 reduction for the patient group with paresis in comparison to the other groups (Figure 5C), while there was no difference between healthy subjects and patients without paresis. For ITC2, there was even an increase of the ITC in patients without paresis in comparison to healthy subjects (Figure 5D). There was no significant correlation of the ERSP and ITC values to age, tumor volume, ADC values, or FA values (*p* > 0.05; Pearson's).

## Discussion

The aim of the present study was to describe the time-frequency representation of MEPs in healthy subjects and brain tumor patients. MEPs triggered by TMS are projecting to a frequency band between 30 and 300 Hz with a local maximum around 100 Hz for both healthy subjects and patients. However, healthy subjects and patients differ in their power and ITC values, although there were no significant differences in the standard time series values of MEPs (i.e., peak-to-peak amplitudes and onset latencies). There was a significant power reduction for higher frequencies between 100 and 200 Hz in patients in contrast to healthy subjects, independent of their current motor status. This “deceleration” of the MEPs was reflected in an increase of the inter-peak latencies of the MEP time series. However, patients with an apparent paresis (MRCS<5) showed an additional disturbance in phase synchronization at these frequencies. In contrast, patients without motor signs did not experience a reduction in ITC during the MEP onset despite exerting a power reduction. Actually, there was an increased ITC during the later phase of the MEP. Since there was no correlation between the CST integrity (as measured by DTI) and the MEP representation in the frequency domain, we hypothesize that differences might have a cortical source, e.g., due to a disturbance of cortical physiology by the brain tumor.

An increase of MEP latency and a decrease of MEP amplitude are generally accepted to indicate a lesion to the corticospinal network (13–15). In brain tumor patients, however, MEP time domain characteristics (i.e., amplitudes and latencies) often do not differ between the lesioned and non-lesioned hemisphere and are similar to those of healthy subjects (11). Comparable to healthy subjects, MEP characteristics in brain tumor patients exert a high inter-subject and intra-subject variability, which has been related to different individual factors such as gender, body height, and antiepileptic drug intake (44). We observed no difference in MEP latencies between healthy subjects and patients. Even in patients with an apparent paresis, there was no significant increase in MEP latencies. Notably, only a significant “deceleration” of the MEP slope was detected for brain tumor patients.

For the time-frequency domain, the present study reveals a projection of MEPs to a frequency band between 30 and 200 Hz for both healthy subjects and patients. This is expected, when considering the MEP peaks (after 27 and 32 ms) as crest and trough of a sine wave with a half wavelength of 5 ms. This data is in good agreement with prior studies evaluating the time-frequency representation of MEPs in animals and humans (22, 26). In patients with brain tumors, high frequencies (>100 Hz) were reduced in comparison to healthy subjects. This represents the frequency equivalent of the MEP deceleration seen in the time series analysis. At the same time, we observed a reduction of inter-trial-coherence in brain tumor patients as a sign of temporal distortion of the MEPs. Notably, ITC changes were most prominent in patients with an apparent paresis. In patients without motor signs, the deceleration of MEPs (as seen in the time series characteristics and the time-frequency representation) resulted in an increase of ITC behind time.

TMS is mediating MEPs by direct (D waves) and transsynaptic activation (I waves) of pyramidal cells (3). As there was no correlation between the CST integrity (as measured by DTI parameter) and the MEP changes in these patients, we hypothesize that the observed differences in the time-frequency domain might have a cortical source, e.g., due to a disturbance of cortical physiology by the brain tumor. We hypothesize that brain tumors are usually diagnosed prior to the invasion of the CST. Thus, in contrast to spinal lesions or strokes, corticospinal transmission of D-waves might be unaffected resulting in regular MEP latencies. The activation of later I-waves produces a sequence of EPSPs that temporally summate and determine the MEP amplitudes albeit arriving at the motoneuron with a longer latency than the initial D-wave (45). Following this line of argumentation, the reduction of MEP amplitude and power in these patients could be attributed to a reduced transsynaptic recruitment of pyramidal cells in the I-wave generation. This could explain the increase of RMT, the MEP amplitude reduction, the decelerated rise of the MEP, and the temporal distortion of MEPs as seen in the ITC analysis. Temporal distortion can be attributed to the failure of TMS to recruit I-waves in brain tumor patients. In patients without motor signs, I-waves might be delayed but still recruitable by TMS. However, it remains unclear why TMS fail to recruit the pyramidal cells, e.g., compression effect of the tumor, oedema, or antiepileptic drug intake.

### Methodological Considerations

To our knowledge, the present work is one of few studies evaluating the MEPs in the frequency domain (26). Although time-frequency methods are very common in the field of neuroscience (6, 16–18), they have not frequently been applied for the evaluation of MEPs. In addition to the need for advanced calculations, there are methodological aspects to be considered. Time-frequency analysis of fast alternating potentials (e.g., MEPs, ECG signals or ripples) are challenged by an apparent, sampling rate dependent, discontinuity of the signal in the time series. Transforming these signals into the frequency domain may cause ringing, a broad band power increase known as “leakage effect.” The amount of spectral leakage depends on the amplitude of the discontinuity. As the discontinuity becomes larger, spectral leakage increases. Thus, fast rising signals like MEPs are very prone to this problem (46).

Time-frequency analysis of digitized signals is traditionally performed using the short-time Fourier transform, which computes the power spectra on successive sliding windows. Long windows provide good frequency resolution and reduce the leakage phenomenon. However, they result in a poor temporal resolution and a “smearing” of the event-related spectral perturbation beyond the actual limits of the time series event. Shortening the window will results in a degradation of frequency resolution with a strong leakage effect (46, 47). Continuous-wavelet transformations such as the Morlet wavelet were introduced to overcome this limitation. The wavelet analysis provides a better temporal resolution by compression/dilation of a mother wavelet as a function of frequency (47). Detecting oscillation packets in time, wavelet techniques seem to be more appropriate to describe MEPs. However, very short wavelets are struggling to distinguish high frequencies (47). Thus, shortening the wavelet length in high frequencies will “smear” the event-related spectral perturbation in a wide range of high frequencies. Balancing these drawbacks, we applied a Morlet wavelet analysis with fixed wavelet length, defined by the observed MEP duration in the time series (i.e., ~40 ms). This enabled an adequate representation of the MEP in relation to the temporal and spectral resolution. However, one has to take into account that frequencies with wave lengths longer than the wavelet are not detectable (here below 25–30 Hz) and that phase detection is inaccurate in higher frequencies. Apart from these time-frequency decomposition methods, time-frequency representation can also be obtained by fitting an autoregressive (AR) model to the signal (48). This approach is very common in ECG analysis (49); however, it is strongly affected by the signal-to-noise ratio (48). Thus, it could be insufficient in situations with small MEP amplitudes such as stroke or brain tumors. Up to date, it remains unclear which method is most suitable for the time-frequency transformation of MEPs. Studies analyzing the time-frequency representation of somatosensory potentials in humans have used both a Fourier transformation (22, 25) and a Morlet wavelet approach (26).

### Limitations of the Study

There are several limitations of the study that should be addressed. Although there was no statistical difference between the healthy subject group and the patient group without any motor signs, there was no good age-matched control. As age and related medical complaints (e.g., diabetes) are known to affect corticospinal conduction and MEP latencies, it cannot be completely excluded that temporal dispersion observed in Group 3 may be attributed to the higher age of the patients. Furthermore, there would be a special interest in the MEPs of the unaffected hemisphere in these patients to avoid potential biases related to a control group. Such an analysis could unravel the effect of individual but tumor-unrelated factors on MEP inter-trial-coherence (e.g., antiepileptic drug intake). Concluding, after introduction of the mentioned approach, further studies with a larger patient group and age-matched comparison cohort are necessary to confirm the described findings. Furthermore, it has to be mentioned that the current analysis includes MEPs elicited after stimulation of different brain areas (e.g., primary motor cortex and/or premotor areas) and different coil positions. Notably, it is known that slight variations in coil placement may result in different MEP responses (50).

## Conclusion

To the best of our knowledge, this is the first study evaluating MEPs of brain tumor patients in the frequency domain. Our findings demonstrate how time-frequency analysis techniques could provide additional information about the MEP (e.g., shape) and the status of the motor system in brain tumor patients. This first evaluation indicates that brain tumors might affect cortical physiology and the responsiveness of the cortex to TMS, resulting in a temporal dispersion of the corticospinal transmission.

## Data Availability Statement

The raw data supporting the conclusions of this article will be made available by the authors, without undue reservation.

## Ethics Statement

The studies involving human participants were reviewed and approved by local ethics committee of the Eberhardt Karls University Tuebingen. The patients/participants provided their written informed consent to participate in this study.

## Author Contributions

KM contributed to the acquisition, analysis, interpretation of data, and writing of the first draft. ALG, MTL, ML, LT, SW, and MT contributed to the data acquisition, interpretation of data, and the review and critique of the final manuscript. AG and MT contributed to the interpretation of data and the review and critique of the final manuscript. GN was responsible for the conception and design, data acquisition, analysis, and interpretation as well as the review and critique of the manuscript. All authors contributed to the article and approved the submitted version.

## Conflict of Interest

The authors declare that the research was conducted in the absence of any commercial or financial relationships that could be construed as a potential conflict of interest.

## Abbreviations

ADC: apparent diffusion coefficient
Amp: amplitude
APB: abductor pollicis brevis muscle
AR: autoregression model
DTI: diffusion tensor imaging
EMG: electromyography
ERSP: event-related spectral perturbation
EP: evoked potentials
FA: fractional anisotropy
FDI: first dorsal interosseous muscle
ITC: inter-trial coherence
Lat: latency
MEP: motor-evoked potential
MRCS: medical research council scale
nTMS: navigated transcranial magnetic stimulation
RMT: resting motor threshold
ROI: region of interest.

## References

[1] Stinear CM Barber PA Smale PR Coxon JP Fleming MK Byblow WD. Functional potential in chronic stroke patients depends on corticospinal tract integrity. Brain. (2007) 130:170–80. 10.1093/brain/awl333 17148468

[2] Chen R Cros D Curra A Di Lazzaro V Lefaucheur JP Magistris MR. The clinical diagnostic utility of transcranial magnetic stimulation: report of an IFCN committee. Clin Neurophysiol. (2008) 119:504–32. 10.1016/j.clinph.2007.10.014 18063409

[3] Rossini PM Burke D Chen R Cohen LG Daskalakis Z Di Iorio R. Non-invasive electrical and magnetic stimulation of the brain, spinal cord, roots and peripheral nerves: basic principles and procedures for routine clinical and research application: an updated report from an I.F.C.N. Committee. Clin Neurophysiol. (2015) 126:1071–107. 10.1016/j.clinph.2015.02.001 25797650 PMC6350257

[4] Groppa S Oliviero A Eisen A Quartarone A Cohen LG Mall V. A practical guide to diagnostic transcranial magnetic stimulation: report of an IFCN committee. Clin Neurophysiol. (2012) 123:858–82. 10.1016/j.clinph.2012.01.010 22349304 PMC4890546

[5] Darling WG Wolf SL Butler AJ. Variability of motor potentials evoked by transcranial magnetic stimulation depends on muscle activation. Exp Brain Res. (2006) 174:376–85. 10.1007/s00221-006-0468-9 16636787 PMC3582032

[6] Naros G Lehnertz T Leão MT Ziemann U Gharabaghi A. Brain state-dependent gain modulation of corticospinal output in the active motor system. Cereb Cortex. (2019) 30:371–81. 10.1093/cercor/bhz093 31204431

[7] van Elswijk G Maij F Schoffelen J-MM Overeem S Stegeman DF Fries P. Corticospinal beta-band synchronization entails rhythmic gain modulation. J Neurosci. (2010) 30:4481–8. 10.1523/JNEUROSCI.2794-09.2010 20335484 PMC6634500

[8] Khademi F Royter V Gharabaghi A. Distinct beta-band oscillatory circuits underlie corticospinal gain modulation. Cereb Cortex. (2018) 28:1502–15. 10.1093/cercor/bhy016 29415124 PMC6093341

[9] Wolf SL Butler AJ Campana GI Parris TA Struys DM Weinstein SR. Intra-subject reliability of parameters contributing to maps generated by transcranial magnetic stimulation in able-bodied adults. Clin Neurophysiol. (2004) 115:1740–7. 10.1016/j.clinph.2004.02.027 15261852

[10] Sollmann N Wildschuetz NN Kelm A Conway N Moser T Bulubas L. Associations between clinical outcome and navigated transcranial magnetic stimulation characteristics in patients with motor-eloquent brain lesions: a combined navigated transcranial magnetic stimulation–diffusion tensor imaging fiber tracking approach. J Neurosurg. (2017) 128:800–10. 10.3171/2016.11.JNS162322 28362239

[11] Picht T Strack V Schulz J Zdunczyk A Frey D Schmidt S Assessing the functional status of the motor system in brain tumor patients using transcranial magnetic stimulation. Acta Neurochir. (2012) 154:2075–81. 10.1007/s00701-012-1494-y 22948747

[12] Butler AJ Kahn S Wolf SL Weiss P. Finger extensor variability in TMS parameters among chronic stroke patients. J Neuroeng Rehabil. (2005) 2:10. 10.1186/1743-0003-2-10 15927075 PMC1175099

[13] Cirillo J Calabro FJ Perez MA. Impaired organization of paired-pulse TMS-induced I-waves after human spinal cord injury. Cereb Cortex. (2016) 26:2167–77. 10.1093/cercor/bhv048 25814508 PMC4830292

[14] Kobayashi M Pascual-Leone A Transcranial magnetic stimulation in neurology. Lancet Neurol. (2003) 2:145–56. 10.1016/S1474-4422(03)00321-1 12849236

[15] Hallett M Transcranial magnetic stimulation and the human brain. Nature. (2000) 406:147–50. 10.1038/35018000 10910346

[16] Naros G Naros I Grimm F Ziemann U Gharabaghi A. Reinforcement learning of self-regulated sensorimotor β-oscillations improves motor performance. Neuroimage. (2016) 134:142–52. 10.1016/j.neuroimage.2016.03.016 27046109

[17] Naros G Grimm F Weiss D Gharabaghi A. Directional communication during movement execution interferes with tremor in Parkinson's disease. Mov Disord. (2018) 33:251–61. 10.1002/mds.27221 29427344

[18] Naros G Gharabaghi A. Physiological and behavioral effects of β-tACS on brain self-regulation in chronic stroke. Brain Stimul. (2017) 10:251–9. 10.1016/j.brs.2016.11.003 27965067

[19] Wang Y Li G Luk KDK Hu Y. Component analysis of somatosensory evoked potentials for identifying spinal cord injury location. Sci Rep. (2017) 7:2351. 10.1038/s41598-017-02555-w 28539587 PMC5443771

[20] Wang Y Zhang Z Li X Cui H Xie X Luk KD-KK. Usefulness of time-frequency patterns of somatosensory evoked potentials in identification of the location of spinal cord injury. J Clin Neurophysiol. (2015) 32:341–5. 10.1097/WNP.0000000000000167 25626775

[21] Wang Y Cui H Pu J Luk KDK Hu Y. Time-frequency patterns of somatosensory evoked potentials in predicting the location of spinal cord injury. Neurosci Lett. (2015) 603:37–41. 10.1016/j.neulet.2015.07.002 26170248

[22] Hu Y Luk KDK Lu WW Holmes A Leong JCY. Prevention of spinal cord injury with time-frequency analysis of evoked potentials: an experimental study. J Neurol Neurosurg Psychiatry. (2001) 71:732–40. 10.1136/jnnp.71.6.732 11723192 PMC1737639

[23] Hu Y Liu H Luk KD. Time-frequency analysis of somatosensory evoked potentials for intraoperative spinal cord monitoring. J Clin Neurophysiol. (2011) 28:504–11. 10.1097/WNP.0b013e318231c15c 21946365

[24] Zhang ZG Yang JL Chan SC Luk KDK Hu Y. Time-frequency component analysis of somatosensory evoked potentials in rats. Biomed Eng Online. (2009) 8:4. 10.1186/1475-925X-8-4 19203394 PMC2669798

[25] Hu Y Luk KDK Lu WW Leong JCY. Application of time-frequency analysis to somatosensory evoked potential for intraoperative spinal cord monitoring. J Neurol Neurosurg Psychiatry. (2003) 74:82–7. 10.1136/jnnp.74.1.82 12486272 PMC1738163

[26] Singh N Saini M Kumar N Deepak KK Anand S Srivastava MVP Time-frequency analysis of motor-evoked potential in patients with stroke vs healthy subjects: a transcranial magnetic stimulation study. SN Compr Clin Med. (2019) 1:764–80. 10.1007/s42399-019-00113-1

[27] Kraus D Naros G Bauer R Leão MT Ziemann U Gharabaghi A. Brain-robot interface driven plasticity: distributed modulation of corticospinal excitability. Neuroimage. (2016) 125:522–32. 10.1016/j.neuroimage.2015.09.074 26505298

[28] Kraus D Gharabaghi A. Projecting navigated TMS sites on the gyral anatomy decreases inter-subject variability of cortical motor maps. Brain Stimul. (2015) 8:831–7. 10.1016/j.brs.2015.03.006 25865772

[29] Mathew J Kübler A Bauer R Gharabaghi A. Probing corticospinal recruitment patterns and functional synergies with transcranial magnetic stimulation. Front Cell Neurosci. (2016) 10:175. 10.3389/fncel.2016.00175 27458344 PMC4932869

[30] Leão MT Naros G Gharabaghi A. Detecting poststroke cortical motor maps with biphasic single- and monophasic paired-pulse TMS. Brain Stimul. (2020) 13:1102–4. 10.1016/j.brs.2020.05.005 32418913

[31] Delorme A Makeig S. EEGLAB: an open source toolbox for analysis of single-trial EEG dynamics including independent component analysis. J Neurosci Methods. (2004) 134:9–21. 10.1016/j.jneumeth.2003.10.009 15102499

[32] Oostenveld R Fries P Maris E Schoffelen J-MM. FieldTrip: open source software for advanced analysis of MEG, EEG, and invasive electrophysiological data. Comput Intell Neurosci. (2011) 2011:156869. 10.1155/2011/156869 21253357 PMC3021840

[33] Grandchamp R Delorme A. Single-trial normalization for event-related spectral decomposition reduces sensitivity to noisy trials. Front Psychol. (2011) 2:236. 10.3389/fpsyg.2011.00236 21994498 PMC3183439

[34] Prentice MJ Fisher NI Statistical analysis of circular data. J R Stat Soc Ser A. (2006) 37:229–30. 10.2307/2983422

[35] Krieg SMSM Buchmann NHNH Gempt J Shiban E Meyer B Ringel F. Diffusion tensor imaging fiber tracking using navigated brain stimulation—a feasibility study. Acta Neurochir. (2012) 154:555–63. 10.1007/s00701-011-1255-3 22270529

[36] Machetanz K Trakolis L Leão MT Liebsch M Mounts K Bender B. Neurophysiology-driven parameter selection in nTMS-based DTI tractography: a multidimensional mathematical model. Front Neurosci. (2019) 13:1373. 10.3389/fnins.2019.01373 31920523 PMC6930230

[37] Rosenstock T Giampiccolo D Schneider H Runge SJ Bährend I Vajkoczy P. Specific DTI seeding and diffusivity-analysis improve the quality and prognostic value of TMS-based deterministic DTI of the pyramidal tract. NeuroImage Clin. (2017) 16:276–85. 10.1016/j.nicl.2017.08.010 28840099 PMC5560117

[38] Weiss C Tursunova I Neuschmelting V Lockau H Nettekoven C Oros-Peusquens AM. Improved nTMS- and DTI-derived CST tractography through anatomical ROI seeding on anterior pontine level compared to internal capsule. NeuroImage Clin. (2015) 7:424–37. 10.1016/j.nicl.2015.01.006 25685709 PMC4314616

[39] Raffa G Scibilia A Germanò A Conti A nTMS-based DTI fiber tracking of motor pathways. In: Navigated Transcranial Magnetic Stimulation in Neurosurgery. Cham: Springer (2017). p. 97–114. 10.1007/978-3-319-54918-7_6

[40] Raffa G Quattropani MC Germanò A. When imaging meets neurophysiology: the value of navigated transcranial magnetic stimulation for preoperative neurophysiological mapping prior to brain tumor surgery. Neurosurg Focus. (2019) 47:9640. 10.3171/2019.9.FOCUS19640 31786549

[41] Weiss Lucas C Tursunova I Neuschmelting V Nettekoven C Oros-Peusquens AM Stoffels G. Functional MRI vs. navigated TMS to optimize M1 seed volume delineation for DTI tractography. A prospective study in patients with brain tumours adjacent to the corticospinal tract. NeuroImage Clin. (2017) 13:297–309. 10.1016/j.nicl.2016.11.022 28050345 PMC5192048

[42] Frey D Strack V Wiener E Jussen D Vajkoczy P Picht T. A new approach for corticospinal tract reconstruction based on navigated transcranial stimulation and standardized fractional anisotropy values. Neuroimage. (2012) 62:1600–9. 10.1016/j.neuroimage.2012.05.059 22659445

[43] Maris E Oostenveld R Non-parametric statistical testing of EEG- and MEG-data. J Neurosci Methods. (2007) 164:177–90. 10.1016/j.jneumeth.2007.03.024 17517438

[44] Sollmann N Bulubas L Tanigawa N Zimmer C Meyer B Krieg SM. The variability of motor evoked potential latencies in neurosurgical motor mapping by preoperative navigated transcranial magnetic stimulation. BMC Neurosci. (2017) 18:4. 10.1186/s12868-016-0321-4 28049425 PMC5209850

[45] Rossini PM Caramia MD Iani C Desiato MT Sciarretta G Bernardi G. Magnetic transcranial stimulation in healthy humans: influence on the behavior of upper limb motor units. Brain Res. (1995) 676:314–24. 10.1016/0006-8993(95)00113-5 7614001

[46] Herrmann CS Rach S Vosskuhl J Strüber D. Time-frequency analysis of event-related potentials: a brief tutorial. Brain Topogr. (2014) 27:438–50. 10.1007/s10548-013-0327-5 24194116

[47] Mallat S A Wavelet Tour of Signal Processing. Burlington, MA: Academic Press (2009). 10.1016/B978-0-12-374370-1.X0001-8

[48] Supp GG Schlögl A Trujillo-Barreto N Müller MM Gruber T. Directed cortical information flow during human object recognition: analyzing induced EEG gamma-band responses in brain's source space. PLoS ONE. (2007) 2:e684. 10.1371/journal.pone.0000684 17668062 PMC1925146

[49] Zhao Q Zhang L ECG feature extraction and classification using wavelet transform and support vector machines. In: Zhao M Shi Z editors. 2005 International Conference on Neural Networks and Brain, Beijing. Piscataway, NJ: IEEE (2005). p. 1089–92. 10.1109/ICNNB.2005.1614807

[50] Bashir S Perez JM Horvath JC Pascual-Leone A. Differentiation of motor cortical representation of hand muscles by navigated mapping of optimal TMS current directions in healthy subjects. J Clin Neurophysiol. (2013) 30:390–5. 10.1097/WNP.0b013e31829dda6b 23912579 PMC3740163

